# OSM-11 Facilitates LIN-12 Notch Signaling during Caenorhabditis elegans Vulval Development 

**DOI:** 10.1371/journal.pbio.0060196

**Published:** 2008-08-12

**Authors:** Hidetoshi Komatsu, Michael Y Chao, Jonah Larkins-Ford, Mark E Corkins, Gerard A Somers, Tim Tucey, Heather M Dionne, Jamie Q White, Khursheed Wani, Mike Boxem, Anne C Hart

**Affiliations:** 1 Massachusetts General Hospital, Center for Cancer Research, Charlestown, Massachusetts, United States of America; 2 Department of Pathology, Harvard Medical School, Boston, Massachusetts, United States of America; 3 Department of Biology, California State University San Bernardino, San Bernardino, California, United States of America; 4 Center for Cancer Systems Biology (CCSB) and Department of Cancer Biology, Dana-Farber Cancer Institute, Boston, Massachusetts, United States of America; 5 Department of Genetics, Harvard Medical School, Boston, Massachusetts, United States of America; University of Cambridge, United Kingdom

## Abstract

Notch signaling is critical for cell fate decisions during development. Caenorhabditis elegans and vertebrate Notch ligands are more diverse than classical *Drosophila* Notch ligands, suggesting possible functional complexities. Here, we describe a developmental role in Notch signaling for OSM-11, which has been previously implicated in defecation and osmotic resistance in C. elegans. We find that complete loss of OSM-11 causes defects in vulval precursor cell (VPC) fate specification during vulval development consistent with decreased Notch signaling. OSM-11 is a secreted, diffusible protein that, like previously described C. elegans Delta, Serrate, and LAG-2 (DSL) ligands, can interact with the lineage defective-12 (LIN-12) Notch receptor extracellular domain. Additionally, OSM-11 and similar C. elegans proteins share a common motif with Notch ligands from other species in a sequence defined here as the Delta and OSM-11 (DOS) motif. *osm-11* loss-of-function defects in vulval development are exacerbated by loss of other DOS-motif genes or by loss of the Notch ligand DSL-1, suggesting that DOS-motif and DSL proteins act together to activate Notch signaling in vivo. The mammalian DOS-motif protein Deltalike1 (DLK1) can substitute for OSM-11 in C. elegans development, suggesting that DOS-motif function is conserved across species. We hypothesize that C. elegans OSM-11 and homologous proteins act as coactivators for Notch receptors, allowing precise regulation of Notch receptor signaling in developmental programs in both vertebrates and invertebrates.

## Author Summary

The classic view of Notch receptor activation involves receptor binding to transmembrane Notch ligands that contain a conserved DSL (Delta, Serrate, and LAG-2) domain. Here, we find that the Caenorhabditis elegans OSM-11 protein is a novel ligand of the well-characterized Notch signal transduction pathway and plays a role in cell fate specification during development. OSM-11 is a secreted, diffusible protein whose loss decreases Notch signaling in vivo. OSM-11 and related C. elegans proteins do not contain a DSL domain, but contain a conserved motif we have named DOS (Delta and OSM-11) that is also found in the extracellular domain of known Notch ligands in organisms other than C. elegans. The functional mammalian homolog of OSM-11 is the secreted protein Deltalike1 (Dlk1), also known as Preadipocyte Factor 1 (PREF1), which plays a poorly defined role in Notch signaling regulating obesity and other developmental decisions. This suggests that Notch ligands are split into two complementary coligand families that act together to regulate Notch signaling in developmental contexts. In addition to regulating development, DOS ligands play roles in osmotic stress and C. elegans behavior, suggesting previously unsuspected roles for Notch signaling across species.

## Abstract 3

The *C. elegans* OSM-11 protein acts with DSL ligands to activate Notch signaling in cell fate specification and defines a conserved family of potential Notch co-ligands.

## Introduction

The Notch signaling pathway is essential for cell fate determination during embryogenesis and postembryonic development in multicellular organisms. Classical Notch signaling begins with activation of the Notch receptor by transmembrane DSL ligands (Delta and Serrate in *Drosophila* or LAG-2 [Lin and Glp-2] in C. elegans [1–3]) expressed on adjacent cells, resulting in proteolytic cleavage of the Notch receptor, internalization of the ligand-receptor complex, and nuclear translocation of the Notch IC (intracellular) domain [4–8]. In the nucleus, the Notch IC domain acts as a transcriptional regulator together with a conserved transcription factor called Su(H) (Suppressor of Hairless) in *Drosophila* and LAG-1 [Lin and Glp-1] in C. elegans [9,10]. The molecular mechanisms of Notch signaling are highly conserved. Vertebrate homologs exist for each of these components in the Notch signaling pathway, and mutations in Notch signaling have been implicated in various developmental disorders, including Alagille and CADASIL [11–14]

In C. elegans, the Notch receptor LIN-12 (Lineage defective-12) plays critical roles in cell fate specification in multiple tissues. The roles of LIN-12 in two steps of vulval development have been particularly well studied. First, LIN-12 is required for cell fate specification of an anchor cell (AC) and a vulval uterine (VU) cell from the descendents of equipotent precursor cells Z1 and Z4 during the L1 larval stage [15–18]. Loss of *lin-12* signaling generally results in the specification of two ACs, whereas increased *lin-12* signaling results in two VU cells. The AC produces a diffusible epidermal growth factor (EGF) signal that induces the primary (1°) cell fate in P6.p, one of six equipotent vulval precursor cells (VPCs) (reviewed in [19]). Additionally, LIN-12 specifies secondary (2°) cell fates of P5.p and P7.p, two VPCs adjacent to P6.p, by antagonizing EGF signaling via lateral inhibition [17,20]. Loss of *lin-12* signaling generally causes VPCs to take on 1° and tertiary (3°) fates, whereas strong *lin-12* gain-of-function alleles cause VPCs to take on 2° fates with consequent changes in the fates of descendent cells that contribute to the adult vulva.

Canonical Notch receptor ligands are exemplified by *Drosophila* Delta, which contains a conserved N-terminal DSL domain originally found in Delta, Serrate, and LAG-2 proteins [2,3,7,21,22]. The DSL domain is followed by a series of EGF repeats and a transmembrane domain. The DSL domain is critical for Notch receptor activation based on tissue culture studies and genetic analysis [23,24], but Notch ligand EGF repeats are also required for Notch receptor activation [25,26]. Numerous Notch ligands containing DSL domains have been identified in various organisms [23,27–32]. C. elegans LAG-2 is a classical Notch ligand containing a canonical DSL domain and transmembrane domain and is essential for LIN-12 activation in vivo in many contexts [21,22].

Although key components in the Notch pathway were identified decades ago in classical genetic studies in *Drosophila* and C. elegans [33,34], additional proteins that play important or redundant roles in Notch signaling have been identified more recently. C. elegans anterior pharynx defective-1 (APX-1) and DSL-1 are DSL domain–containing soluble proteins that function redundantly with LAG-2 during vulval development [35]. Noncanonical ligands for vertebrate Notch receptors have been identified, including Delta/notch-like EGF repeat containing protein (DNER), F3/contactin, and MAGP proteins [36–40], but functional C. elegans homologs of these noncanonical ligands have not been identified. Deltalike 1 (a.k.a., DLK1, fetal antigen 1 [FA1], ZOG, pG2, Preadipocyte Factor 1 [PREF1]) also encodes a putative soluble Notch ligand that lacks a DSL domain [41–43,44]. DLK1 is a paternally imprinted gene with diverse developmental roles. DLK1 knockout mice are growth retarded and obese with eye and skeletal defects [45]. Overexpression of DLK1 due to polar overdominance results in callipyge sheep with muscle overproliferation and decreased adipogenesis [45–47]. Although *Drosophila* lacks a DLK1 homolog, ectopic expression of mammalian DLK1 in *Drosophila* inhibits Notch signaling [48]. DLK1 has multiple mRNA isoforms; some transcripts are translated as membrane-bound proteins with subsequent proteolytic release of the EGF-repeat–containing extracellular domain, while others encode soluble secreted proteins [42,43,49]. DLK1 EGF repeats bind Notch1 EGF repeats in bacterial two-hybrid assays and inhibit activity of a Notch-dependent reporter gene. However, DLK1 inhibits Notch activation by previously described DSL Notch ligands in these same studies [50]. Therefore, a role for DLK1 as a Notch ligand is controversial, given the lack of a canonical DSL domain and the inability of DLK1 to activate vertebrate Notch receptors.

Here, we examine the secreted C. elegans protein, OSM-11. A role for OSM-11 in osmotic sensitivity and defecation was recently described, but the molecular function of these genes was not elucidated in previous studies, and no homologous proteins outside of nematodes were identified [51,52]. We found that OSM-11 and related C. elegans proteins contain a motif found only in known and putative Notch ligands, including Serrate and DLK1. We examined the functional role of *osm-11* in development. We find that *osm-11* increases *lin-12* Notch receptor signaling during vulval cell fate specification. Our results suggest a model in which OSM-11 normally acts with C. elegans DSL ligands to activate Notch receptor signaling in vivo.

## Results

### OSM-11 Is Required for Cell Fate Specification during Vulval Development

**Figure 1:**
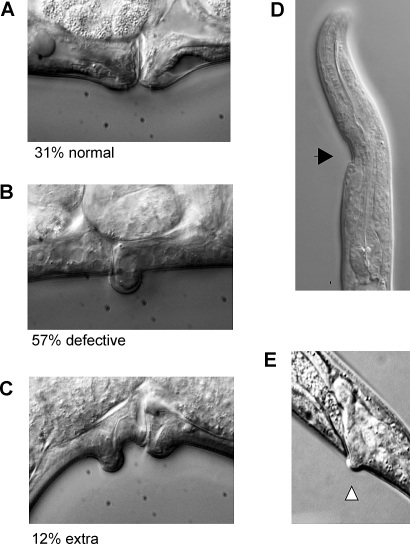
OSM-11 Is Required for Normal Development (A) Thirty-one percent of *osm-11(lf)* adult animals had overtly normal vulva and did not retain eggs resembling control animals. (B) Fifty-seven percent of *osm-11(lf)* animals inappropriately retained eggs and/or had a single misshapen or protruding vulva (15% and 42%, respectively). (C) Twelve percent of *osm-11(lf)* animals had an extra protrusion near the normally positioned vulva. (D) *osm-11(lf)* animals had defects in head morphology at low frequency (arrowhead). (E) Two thirds of *osm-11(lf)* animals had a ventral protrusion behind the anus (arrowhead). *n* > 100 animals were scored.

We identified a deletion allele of *osm-11* that removes all of the predicted mature protein, *osm-11(rt142)*. The majority of animals lacking *osm-11* had visibly misshapen vulva or defective vulva based on retention of eggs (Figure 1A–1C). A smaller fraction had an additional protrusion near the normal position of the vulva. Vulval development was also modestly perturbed by RNA interference (RNAi) knockdown of *osm-11* (16% defective, *n* = 82), suggesting that *osm-11* defects in vulval developmental were caused by loss of *osm-11* function. Consistent with this hypothesis, *osm-11* defects were rescued by reintroduction of either genomic DNA containing the entire *osm-11* gene or the *osm-11* cDNA expressed under the control of 3.4 kb of upstream genomic DNA sequences 5′ to the predicted *osm-11* initiator methionine (described below) and the *unc-54* 3′ UTR. *osm-11* loss of function also caused non-vulval developmental defects, including misshapen heads and anal protrusions (Figure 1D and 1E) reminiscent of animals with decreased Notch signaling or increased EGF signaling [9]. To determine the biochemical role of OSM-11, a molecular and cellular analysis was first undertaken.

### *osm-11* Encodes a Novel Protein with Similarity to Notch Ligands

**Figure 2:**
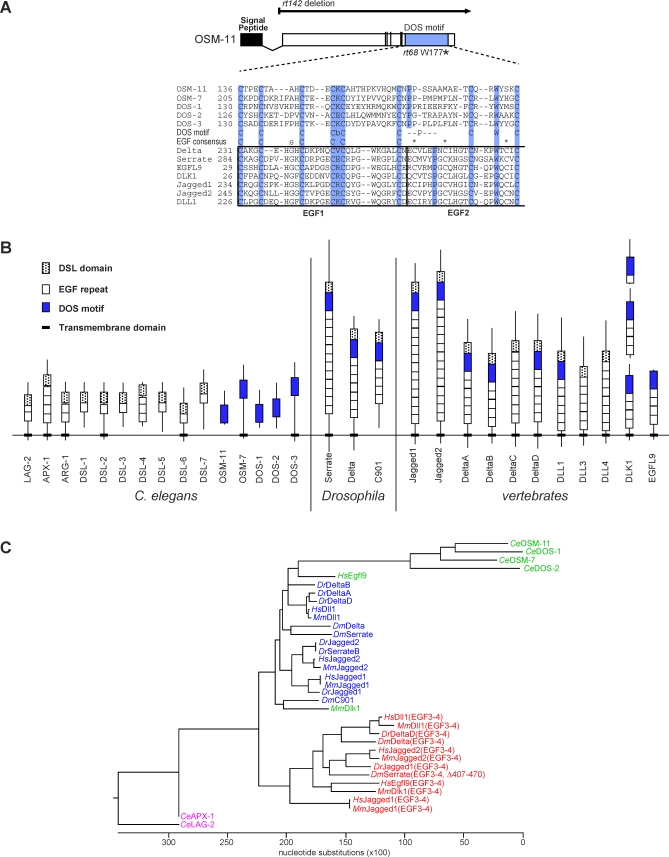
*osm-11* Encodes a Protein with a Conserved Motif Found in Notch Ligands (A) Top: OSM-11 genomic structure. The signal peptide is shaded black, and putative O-linked glycosylation sites are indicated by vertical lines. The DOS motif is shaded blue; it overlaps the previously defined osmotic stress resistant (OSR) motif [52]. *osm-11(rt142)* removes all coding sequence after the signal peptide; *osm-11(rt68)* converts W177 to a premature stop codon. Bottom: the DOS motif-containing sequences from C. elegans OSM-11, OSM-7, DOS-1, DOS-2, and DOS-3 are aligned above the DOS motif consensus and the cEGF-1 consensus [53]. DOS-motif regions from mouse proteins and known *Drosophila* Notch ligands are aligned under the cEGF-1 consensus. DOS-motif amino acids are shaded blue and previously described EGF repeats are boxed. Asterisks (*) indicate cysteines in the conserved EGF-motif that are not found in the C. elegans DOS proteins. The DOS motif consensus is: C-X(3)-C-X(3,8)-C-X(2,5)-C-[KVER]-C-X(10,12)-C-X(1,3)-P-X(6,9)-C-X(1,4)-W-X(1,4)-C. In the DOS motif consensus, b represents K, V, E, or R, and the dash (-) indicates possible positions for proline in the DOS motif. In the cEGF-1 consensus, s represents a small amino acid [53]. (B) The position of the DOS motif in known or predicted C. elegans, *Drosophila*, and vertebrate Notch ligands. The DOS motif overlaps with the first two EGF repeats of canonical Notch ligands and may define a unique subset of EGF repeats. The noncanonical Notch ligands DNER [40], F3/contactin [95], and MAGP [36–40] do not contain a DOS motif (unpublished data). (C) Similarity between DOS motifs, the first and second EGF repeats, and the third and fourth EGF repeats of Notch ligands. As noted by Lissemore and Starmer [27], the first and second EGF repeats differ from the third and fourth EGF repeats. DOS-3 was not included in this alignment. Green indicates the DOS motif of proteins that lack canonical EGF repeats; blue indicates the first and second EGF repeats of Notch ligands; red indicates the third and fourth EGF repeats of Notch ligands; and magenta represents the C. elegans Notch ligands that lack DSL domains. See Materials and Methods for accession numbers and other details.

*osm-11* corresponds to the C. elegans gene designated as F11C7.5 at the National Center for Biotechnology (NCBI). F11C7.5 is predicted to have two exons and one splice form, which was confirmed by cDNA sequencing (unpublished data). OSM-11 and four similar predicted C. elegans proteins, OSM-7 (T05D4.4), ZK507.4, K10G6.2, and K02F3.7, contain a signal peptide for secretion and a potential cEGF-1 domain [53] that is part of a conserved motif described below (Figure 2A). cEGF-1 domains contain a small amino acid and six cysteine residues with characteristic spacing that forms three disulfide bonds, and are found in extracellular proteins including Notch receptors and ligands.

As standard similarity searching programs (i.e., BLAST) failed to identify additional proteins similar to OSM-11 outside of helminthes, we undertook further bioinformatic analysis, which revealed similarity between OSM-11 and previously described Notch ligands. First, the predicted sequences of C. elegans OSM-11, OSM-7, K10G6.2, ZK507.4, and K02F3.7 proteins were aligned, which revealed conserved amino acids in a common motif containing the putative cEGF-1 domain and additional amino acids: C-X(3)-C-X(3,8)-C-X(2,5)-C-[KVER]-C-X(10,12)-C-X(1,3)-P-X(6,9)-C-X(1,4)-W-X(1,4)-C. Motif-based database searches revealed that all proteins containing the new motif in *Drosophila*, zebrafish, mouse, and humans are either DSL-containing Notch ligands or suspected Notch ligands. We named the motif DOS because it is found in Delta and OSM-11-like proteins (shaded in Figure 2) and designated the C. elegans genes ZK507.4, K10G6.2, and K02F3.7 as *dos-1*, *dos-2*, and *dos-3*, respectively. All five C. elegans DOS-motif proteins are likely secreted based on the presence of a predicted N-terminal signal peptide. However, OSM-11 and DOS-3 also have a consensus proprotein convertase protease cleavage site and a C-terminal transmembrane domain, suggesting that they may be translated as transmembrane preproproteins prior to proteolytic processing and release of a soluble DOS protein.

In known Notch ligands from *Drosophila* and vertebrates, the DOS motif is always located immediately following the DSL domain and overlapping the first two EGF repeats. The first two EGF repeats of most Notch ligands differ from the remaining EGF repeats [27] (this study, Figure 2C). The role of these EGF repeats remains unclear, but several previous studies suggest that these EGF repeats play roles in Notch activation: they are required for the DSL domain of Jagged1 to bind to the mammalian Notch2 receptor in biochemical studies [54]; perturbation of the second EGF repeat interferes with Notch signaling in *Drosophila* [25]; and mutations in these EGF repeats of human Jagged1 are associated with Alagille syndrome [55]. The DOS motif may define a unique group of EGF repeats and EGF-like repeats that have a distinct functional role in Notch signaling.

Outside of helminthes, only three proteins were identified with DOS motifs that are not canonical Notch ligands: C901, DLK1, and EGFL9 (DLK2). These proteins have a signal peptide sequence, and the DOS motif is located in the first two EGF repeats (Figure 2B). C901 is a predicted *Drosophila* protein of unknown function containing a DSL domain and multiple EGF repeats [56]; it is unclear whether C901 is a transmembrane DSL domain protein. DLK1 and EGFlike 9 (EGFL9) are vertebrate proteins that contain EGF domains, but lack DSL domains. EGFL9 is poorly characterized [57]. DLK1 has membrane-bound and secreted isoforms, and plays diverse roles in normal development. Altered DLK1 expression causes developmental defects in mammals [42,45–47]. DLK1 EGF repeats containing the DOS motif bind to specific Notch1 receptor EGF repeats in two-hybrid studies and in tissue culture [50], but the role of DLK1 in Notch signaling remains controversial because DLK1 lacks a DSL domain and does not activate mammalian Notch receptors [42,45–47]. Given this controversy and given the limited homology observed between OSM-11 and previously described canonical Notch ligands, we turned to cellular, genetic, and molecular tools in C. elegans to elucidate the role of OSM-11 in developmental signaling pathways.

### Loss of *osm-11* Perturbs Cell Fate Specification during Vulval Development

We first examined the role of *osm-11* in specification of the AC. LIN-12 Notch function is required for cell fate specification of an AC and a VU cell from the equipotent precursor cells Z1 and Z4 during the L1 larval stage [15–18]. Loss of *lin-12* signaling results in the specification of two ACs, whereas increased *lin-12* signaling results in two VU cells. AC cells are readily quantified by examining expression of a *lin-3p::gfp* reporter construct [58]. No alterations in *lin-3p::gfp* were observed in *osm-11(lf)* animals compared to *osm-11(+)* animals (unpublished data; *n* = 92), suggesting that loss of *osm-11* does not alter AC cell fate specification in otherwise normal animals.

**Figure 3:**
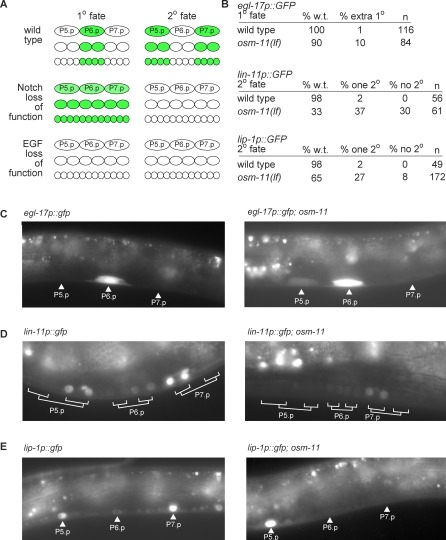
OSM-11 Loss Results in Cell Fate Specification Defects (A) A simplified diagram of cell fate GFP marker expression in P5.p, P6.p, and P7.p. GFP expression is schematically shown in green. Note that equivalence group members P3.p, P4.p, and P8.p are not shown. In wild-type animals, primary (1°) cell fate markers are expressed in P6.p (top left), whereas secondary (2°) cell fate markers are normally expressed in P5.p and P7.p (top right). The first Pn.p division (by P5.p, P6.p, and P7.p) in mid-L3 larvae gives rise to Pn.px cells; the next divisions give rise to Pn.pxx cells in late-L3 larvae. Loss of Notch signaling does not stop 1° cell fate assumption by P6.p, but results in inappropriate adoption of 1° cell fates by P5.p, P7.p, and their descendents. Loss of EGF/Ras signaling results in adoption of the tertiary fate by P5.p and P7.p, and in some cases, P6.p, depending on the severity of the defect [96]. (B) Quantification of data from (C–E). *p* < 0.05 based on χ^2^ for each marker. (C) Ten percent of L3 *osm-11(lf)* animals (right) ectopically express the 1° cell fate marker *egl-17p::gfp* in P5.p or P7.p, which normally adapt the 2° fate (left). (D) Sixty-seven percent of L3 *osm-11(lf)* animals lack expression of the 2° cell fate marker *lin-11p::gfp* in descendants of P5.p and/or P7.p. (E) Thirty-five percent of L3 *osm-11(lf)* animals do not up-regulate expression of the 2° fate marker *lip-1p::gfp* in P5.p and/or P7.p. *p* < 0.05 based on χ^2^ for each marker. These data suggest *osm-11(lf)* animals have a loss of 2° cell fate specification consistent with loss of LIN-12 Notch signaling. In (C–E), arrowheads indicate the positions of P5.p, P6.p, and P7.p.

We next examined VPC specification. After AC specification, the AC produces the diffusible EGF protein LIN-3 that is required for induction of the 1° cell fate in P6.p, one of six equipotent VPCs (reviewed in [19]). LIN-3 EGF acts via the well-characterized Ras/MAPK (mitogen activated protein kinase) pathway in VPCs. LIN-12 Notch function is required to specify 2° cell fates of P5.p and P7.p, two VPCs adjacent to P6.p, by antagonizing EGF signaling via lateral inhibition [17,59]. Loss of EGF signaling eliminates 1° and 2° cell fates, whereas aberrantly increased EGF/Ras/MAP kinase signaling can cause all VPCs to adopt the 1° cell fate. By contrast, loss of *lin-12* Notch signaling causes all VPCs to take on 1° or 3° fates, whereas strong Notch gain-of-function alleles cause all six VPCs to take on 2° fates (Figure 3A). These VPC fate decisions were assessed in *osm-11(lf)* animals and control animals at specific larval stages using the previously described green fluorescent protein (GFP) reporter constructs *egl-17p::gfp*, *lin-11p::gfp*, and *lip-1p::gfp* [60].

In L3 animals, *egl-17p::gfp* expression in P6.p is directly dependent on EGF/Ras signaling, and *egl-17* expression is repressed in P5.p or P7.p by lateral inhibition via LIN-12 Notch signaling [60]. At the Pn.p stage, when cell fates are first established, *egl-17p::gfp* is only expressed in P6.p in wild-type animals. We found appropriate *egl-17p::gfp* expression in the P6.p cell (Figure 3B and 3C) of animals lacking *osm-11*, but ectopic *egl-17p::gfp* expression in P5.p or P7.p in approximately 10% of *osm-11(lf)* L3 animals. This ectopic *egl-17p::gfp* expression suggests that in *osm-11(lf)* animals, P5.p and P7.p secondary cell fates are not correctly established whereas the 1° cell fate choice of P6.p is unaffected. Later, at the L4 larval stage, *egl-17* expression normally is lost in wild-type animals from P6.p descendents and observed only in 2° cell lineages, i.e., in P5.p and P7.p descendants. In 71% of *osm-11(lf)* L4 animals, *egl-17p::gfp* expression in P5.p and/or P7.p descendants was lost, consistent with loss of 2° cell fates (unpublished data; *n* = 63). The aberrant *egl-17p::gfp* expression observed in *osm-11(lf)* animals suggests that 1° and 2° cell fates are not correctly specified in a fraction of *osm-11(lf)* animals, consistent with decreased Notch signaling.

To determine whether secondary cell fates are lost in *osm-11(lf)* animals, cell fate specification was examined using *lin-11p::gfp* and *lip-1p::gfp* reporter genes. *lin-11p::gfp* is expressed exclusively in P5.p and P7.p vulval secondary lineages during development [61,62] (98% of control wild-type late-L3 larvae), but *lin-11p::gfp* expression is lost in P5.p and/or P7.p descendents in 67% of *osm-11(lf)* animals (Figure 3B and 3D). Strikingly, 69% of *osm-11(lf)* adult animals had an overtly defective vulva or retained eggs (Figure 1), which correlates quantitatively with the loss of secondary cell fates observed with altered *lin-11p::gfp* expression. Loss of *lin-11* expression at this stage suggests that secondary cell fates are either not properly specified or not maintained in *osm-11(lf)* animals.

Secondary cell fate specification can be more directly assessed using *lip-1p::gfp*. In normal L3 animals, *lip-1p::gfp* expression is up-regulated in P5.p and P7.p upon assumption of secondary cell fate [63]. This up-regulation is directly dependent on *lin-12* Notch receptor signaling. However, in 35% of *osm-11(lf)* L3 animals, *lip-1p::gfp* was not up-regulated in P5.p and/or P7.p (Figure 3B and 3E; vs. up-regulation in 98% of control animals). The loss of *lip-1p::gfp* and *lin-11p::gfp* expression observed in *osm-11(lf)* animals is reminiscent of changes observed when LIN-12 Notch signaling is decreased and is not consistent with decreased EGF/Ras signaling.

### *osm-11* Is Expressed in VPCs and Hypodermal Cells

**Figure 4:**
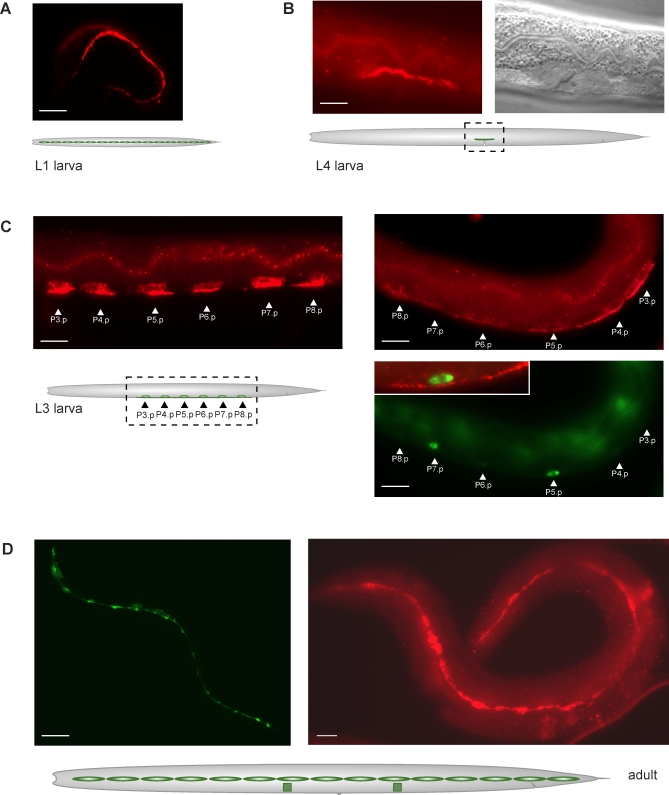
*osm-11* Is Expressed in VPCs and Other Tissues (A) OSM-11 expression in seam cells of L1 larvae detected using α-OSM-11 antisera. The seam cells on the right side of an L1 animal are in focus; the seam cells on the left side are visible and slightly out of focus. OSM-11 was not expressed in seam cells or hypoderm at other larval stages. (B) OSM-11 expression in the developing uterus of L4 larvae. Left, α-OSM-11 antisera staining; right, visible light image. (C) OSM-11 expression in vulval precursor cells (VPCs; arrowheads) in L3 larvae. The top panels show α-OSM-11 antisera staining of VPCs prior (top left) and immediately after (top right) cell fate specification as assessed by *lip-1p::gfp* expression. An overlay of α-OSM-11 staining and *lip-1p::gfp* expression shows that OSM-11 is concentrated on the apical surface of the VPCs (bottom right); this was confirmed using an *ajm-1::gfp* fusion (unpublished data). (D) OSM-11 expression in seam cells and spermatheca in adult animals. An *osm-11p::gfp* reporter gene containing *unc-54* 3′ UTR sequences is expressed in adult seam cells (left); α-OSM-11 antisera was used to confirm seam cell and spermatheca expression (right). No OSM-11 was detected in neurons of larvae or adult animals (unpublished data); embryonic expression was not characterized. In (A–D), the scale bar represents 10 μm.

The functional significance of the similarity of OSM-11 to classic Notch ligands was unclear, particularly as OSM-11 lacks a DSL domain. To address the role of *osm-11* in development, the cellular and temporal pattern of *osm-11* expression was examined to delineate its potential roles in VPC fate specification. A transcriptional GFP reporter (*osm-11p::gfp*) was generated using the same upstream sequences used for *osm-11* cDNA rescue. In animals harboring this transgene, GFP expression was observed in numerous unidentified cells during embryonic development from the comma stage onward (unpublished data). GFP expression was observed in the VPCs during larval development, as well as various hypodermal cells during larval stages (Figure 4). Using polyclonal antisera raised against OSM-11 to stain wild-type animals, we found that OSM-11 was expressed in the VPCs of L3 larvae prior to and during cell fate specification (Figure 4B). OSM-11 immunoreactivity was also observed in the seam cells of L1 larvae and adult animals (Figure 4A and 4D). In adult animals, *osm-11p::gfp* was expressed only in hypodermal seam cells in adult animals; hypodermal seam cell expression in adult animals was also confirmed with staining with OSM-11 antisera (Figure 4D). The larval hypodermal expression pattern of *osm-11p::gfp* is reminiscent of the *osm-7p::gfp* expression pattern described previously, but *osm-7p::gfp* expression in seam cells was not reported [52]. OSM-11 protein was also expressed in the developing uterus of L4 larvae (Figure 4B) and in the spermatheca (Figure 4D); the LIN-12 Notch receptor plays a developmental role in these tissues as well [64], but only OSM-11 expression in VPCs was characterized further.

Initially, OSM-11 protein is detected at uniform levels in all six equivalent VPCs. OSM-11 disappears from P5.p, P6.p, and P7.p after 1° and 2° vulval cell fates are specified (based on up-regulation of *lip-1p::gfp*; Figure 4B). OSM-11 was not detected in VPC descendents. Previously described C. elegans DSL-containing Notch ligands also have temporally regulated expression patterns in the VPCs [35]. For example, based on reporter construct analysis, soluble DSL-1 is only expressed in P6.p and its descendents. OSM-11 expression in Pn.p cells is consistent with a role for OSM-11 in initial cell fate specification.

Like LIN-12 Notch receptors, OSM-11 is primarily localized to the apical side of VPCs (Figure 4B, inset). VPCs are polarized epithelial cells; EGF and Notch signaling normally occurs in separate cellular compartments. Lethal-23 (LET-23) EGF receptors are localized to the basolateral surface of the VPCs in close proximity to the AC [65], which is the source of LIN-3 EGF. In contrast, LIN-12 receptors are primarily localized to the apical surface of the VPCs. The apical localization of OSM-11 in VPCs during cell fate specification suggests that OSM-11 is available to bind to LIN-12 receptors in VPCs at the time of cell fate specification.

### Osmotic Stress Response Does Not Alter Vulval Cell Fate Specification

*osm-11* and *osm-7* were previously implicated in osmotic stress resistance [51,52]. Pre-exposure of wild-type C. elegans to high external osmolarity is sufficient to induce osmotic resistance. Loss of either *osm-7* or *osm-11* allows animals to survive high external osmolarity without pre-exposure. The cellular and molecular mechanisms underlying osmotic stress resistance in either scenario are poorly understood, but up-regulation of *gpdh-1* and increased levels of the osmolyte glycerol have been implicated [51,52]. As loss of *osm-11* increases glycerol levels and increased osmolyte levels can alter protein folding, *osm-11* could act indirectly to decrease Notch receptor signaling in VPC fate specification. Alternatively, OSM-11 might act directly upon Notch receptors involved in VPC fate specification. Our experimental results below favor the latter model; the role of OSM-11 in vulval cell fate specification is distinct from the role of OSM-11 in osmotic stress.

If osmotic stress indirectly decreases Notch receptor signaling, then vulval development should be altered by osmotic stress and altered by genetic backgrounds with increased osmotic stress resistance. We first tested this hypothesis by raising wild-type animals under previously defined osmotic stress conditions: 200 and 400 mM NaCl. Rearing under osmotic stress conditions did not alter vulval morphology, and the cellular expression patterns of vulval cell lineage markers (*lip-1p::gfp*, *egl-17p::gfp*, or *lin-11p::gfp*) in VPCs were unchanged (unpublished data). We also examined genetic backgrounds previously implicated in osmotic stress resistance; neither *osr-1* nor *daf-2* animals have altered vulval morphology [66–68]. In addition, we considered the possibility that OSM-11 expression in the vulval cell precursors might be altered by osmotic stress. We found that rearing under osmotic stress conditions (400 mM NaCl) did not alter OSM-11 protein levels in VPCs (unpublished data). Combined, all of these data suggest that osmotic stress does not itself regulate vulval development. Instead, these data suggest that the roles of *osm-11* in vulval development and osmotic stress resistance are independent.

### OSM-11 Is a Secreted Protein

**Figure 5:**
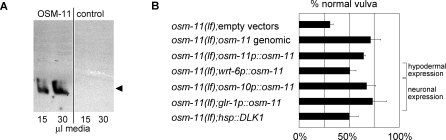
*osm-11* Encodes a Secreted Protein Required for Vulval Development (A) Western blot of conditioned media from *Drosophila* S2 cells containing an OSM-11 cDNA expression construct or empty vector. OSM-11 was not detected in cell lysates (unpublished data). The molecular weight of mature OSM-11 was predicted at 18.9 kDa; the detected protein migrated at 20.7 kDa (arrowhead). OSM-11 may be O-linked glycosylated (see Figure 2). (B) Transgenic rescue of *osm-11(lf)* vulval defects. *osm-11(lf)* animals harboring transgenes with empty expression vectors were indistinguishable from nontransgenic *osm-11(lf)* animals (*n* = 129 animals, 5 transgenic lines) and were used as controls. Multiple transgenic lines were scored for all rescue experiments; data are reported as mean ± standard error of the mean (S.E.M.) In addition to a genomic *osm-11* construct, expression of the *osm-11* cDNA using the following promoters also significantly rescued *osm-11(lf)* vulval defects: *osm-11p*, *hsp-16p* (ubiquitous expression; 79% normal vulval; unpublished data), *wrt-6p* (hypodermal), *osm-10p* (sensory neurons), and *glr-1p* (nonoverlapping set of neurons vs. *osm-10p*). In addition, heterologous expression of mammalian DLK1 driven by the *hsp-16* promoter also significantly rescued *osm-11(lf)* vulval phenotypes. *n* > 52 animals for each transgene, *p* < 0.05 by χ^2^.

Because the predicted peptide sequence of OSM-11 contains a signal peptide, we tested whether OSM-11 is a secreted protein. When an *osm-11* cDNA was expressed in *Drosophila* S2 tissue culture cells, OSM-11 protein accumulates in the media and not in cells (Figure 5A), consistent with OSM-11 acting in vivo as a soluble protein in the extracellular milieu. The ability of OSM-11 to diffuse and act as a soluble factor in vivo was tested by ectopically expressing OSM-11 in non-VPC cells in *osm-11(lf)* animals. *osm-11* cDNA was fused to *osm-10* or *glr-1* promoter fragments that drive expression in nonoverlapping subsets of neurons throughout larval development. The *osm-10* promoter drives expression in four classes of sensory neurons located exclusively in the head and tail [69]. The *glr-1* promoter drives expression in 17 other classes of neurons (distinct from *osm-10*–expressing neurons) located in the head and tail [70,71]. Some of the *glr-1*–expressing neurons have processes in the ventral nerve cord near the VPCs. We found that neuronal expression of the *osm-11* cDNA significantly rescued *osm-11* vulval defects to levels comparable with *osm-11* promoter-driven cDNA rescue (Figure 5B). Consistent with these results, hypodermal expression of *osm-11* cDNA using the *wrt-6* promoter [72] also rescued *osm-11* defects, albeit at a lower level. We conclude that *osm-11* can act nonautonomously and that soluble OSM-11 can diffuse in vivo. Although OSM-11 expressed in VPCs may be sufficient for normal vulval development, OSM-11 can probably function at a distance in some contexts like soluble DSL ligands in C. elegans [35].

### *osm-11* Acts Upstream of *lin-12* Notch Receptor Activation to Increase Signaling

The phenotypic defects caused by loss of *osm-11* might be due to OSM-11 action upon previously identified molecular pathways that regulate cell fate specification in vulval development. We tested the sensitivity of the EGF, Notch, and synthetic multivulva (SynMuv) VPC fate specification pathways to *osm-11* levels by RNAi knockdown of *osm-11* in mutants that have been previously used as sensitized backgrounds for each pathway: *lin-12(n137n460csgf)* Notch (see below), *let-23(sa62gf)* EGF receptor, *let-60(n1046gf)* Ras, or *lin-15(n765tslf)* SynMuv [73–78]. *osm-11(RNAi)* had the most effect in animals with compromised *lin-12* Notch signaling (27% change in multivulva (Muv) of *lin-12(n137n460);osm-11(RNAi)* at 20 °C versus less than 9% change in other backgrounds, *p* < 0.05, *n* > 50 each). Although it is difficult to assess the relative sensitivity of these various genetic backgrounds, these results suggested that Notch signaling might be particularly sensitive to OSM-11 levels and that *osm-11* might modulate *lin-12* signaling during vulval development.

**Figure 6:**
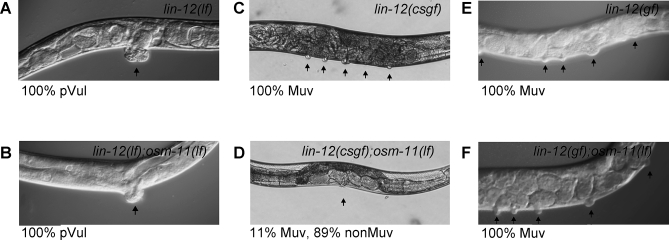
*osm-11* Normally Increases Notch Signaling during Vulval Development (A and B) *lin-12(lf)* is epistatic to *osm-11(lf)*. *lin-12(lf)* is the null allele *n941*; animals carrying this allele have a protruding vulva (pVul; [A]) that is distinct from the defective vulva seen in *osm-11(lf)* animals (see Figure 1). *lin-12(lf);osm-11(lf)* animals were indistinguishable from *lin-12(lf)* animals (B). (C and D) *osm-11(lf)* suppresses *lin-12(csgf)* at 15 °C. *lin-12(csgf)* is *n137n460*, a recessive cold-sensitive gain-of-function allele; animals carrying this mutation have multiple pseudovulvae (Muv; [C]). *lin-12(csgf);osm-11(lf)* animals were significantly less Muv (nonMuv) than *lin-12(csgf)* animals ([D]; *p* < 0.05). (E and F) *osm-11(lf)* does not suppress *lin-12(gf)*. *lin-12(gf)* is *n137*, a dominant gain-of-function allele that is ligand independent; animals carrying this mutation are Muv (E). *lin-12(gf);osm-11(lf)* animals were indistinguishable from *lin-12(gf)* animals (F). *n* > 50 animals were scored for each genotype.

To more accurately assess the possible role of *osm-11* in *lin-12* Notch signaling in vivo, we undertook genetic studies using the *osm-11(lf)* null allele and previously described *lin-12* alleles. *lin-12(n137)* is a ligand-independent dominant gain of function (*gf*) allele, whereas *lin-12(n137n460)* is a recessive, cold-sensitive gain-of-function allele (*csgf*). Both cause multiple ectopic vulvae (Muv) due to secondary cell specification defects [79,80]. If OSM-11 normally functions to increase Notch signaling, then loss of OSM-11 should decrease LIN-12 Notch signaling. We found that *osm-11(lf)* partially suppressed the Muv defect of *lin-12(csgf)* at the restrictive temperature, consistent with OSM-11 normally increasing *lin-12* signaling (Figure 6C and 6D). However, *osm-11(lf)* did not suppress the stronger *lin-12(gf)* allele (Figure 6E and 6F). Since *lin-12(n137gf)* is thought to activate *lin-12* signaling in a ligand-independent manner, the inability of *osm-11(lf)* to suppress *lin-12(gf)* is consistent with *osm-11* acting before or during ligand activation of LIN-12.

If *osm-11* normally acts before or during ligand activation of LIN-12, then *lin-12(lf)* should be epistatic to *osm-11(lf)*. *lin-12(lf)* animals are sterile and have a single large protruding vulva [80], a phenotype that is easily distinguishable from the misshapen vulva of *osm-11(lf)* animals (compare Figure 1B and 1C with Figure 6B). *lin-12(lf);osm-11(lf)* double-mutant animals were indistinguishable from *lin-12(lf)* animals, suggesting that *osm-11* acts upstream of *lin-12* Notch (Figure 6A and 6B). Combined, these results suggest that OSM-11 normally increases LIN-12 Notch signaling in vivo and acts before or during receptor activation.

### OSM-11 Functions with Other DOS Proteins in Development

Five C. elegans genes encode putative secreted DOS-motif proteins: *osm-11*, *osm-7*, *dos-1*, *dos-2* [51,52], and *dos-3*. Loss-of-function alleles are not currently available for *dos-*2 and *dos-3*, but *osm-7(tm2256)* and *dos-1(ok2398)* are deletion alleles generated by the C. elegans gene knockout consortia and are likely strong loss-of-function (lf) or null alleles. *osm-7(tm2256lf)* animals are resistant to osmotic stress and fail to avoid high osmolarity, similar to previously published *osm-7* alleles [51,52].

**Figure 7:**
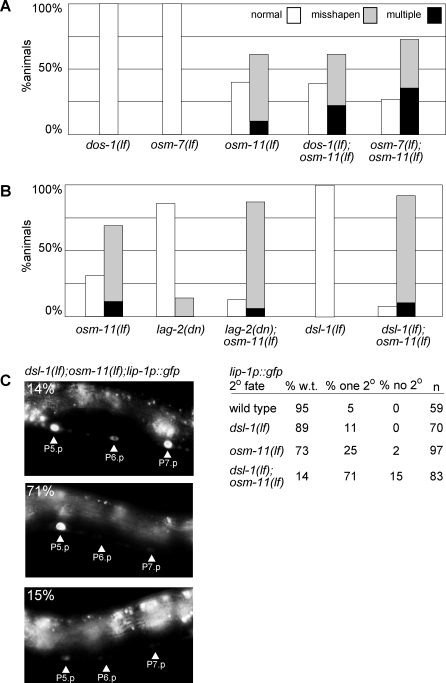
OSM-11 Acts Synergistically with DSL Ligands and Other DOS Proteins In (A and B), phenotypes were scored as in Figure 1. (A) Genetic interactions between *osm-11* and DOS-motif genes *osm-7* and *dos-1* (ZK507.4). *dos-1(lf)* and *osm-7(lf)* are both presumptive null alleles, and animals harboring these alleles had normal vulvas. *dos-1(lf);osm-11(lf)* and *osm-7(lf);osm-11(lf)* animals had significantly more severe defects than *osm-11(lf)* animals (*p* < 0.005, χ^2^ test). Mutant alleles of *dos-2* (K10G6.2) and *dos-3* (K02F3.7) are not currently available. (B) Genetic interactions between *osm-11* and DSL-domain genes *lag-2* and *dsl-1*. *lag-2(dn)* is the dominant negative allele *sa37*; *dsl-1(lf)* is *ok810* and is a presumptive null allele. *lag-2(dn)* and *dsl-1(lf)* animals had few or no vulval defects. *lag-2(dn);osm-11(lf)* and *dsl-1(lf);osm-11(lf)* animals had significantly more-severe defects that *osm-11(lf)* animals (*p* < 0.005, χ^2^ test). (C) Vulval precursor cell (VPC) fate analysis for *osm-11* and *dsl-1*. Arrowheads indicate the positions of P5.p, P6.p, and P7.p. Secondary (2°) cell fates were scored as in Figure 3 using *lip-1p::GFP* as illustrated (right). *dsl-1;osm-11* double-mutant animals had significantly more severe 2° fate specification defects compared to either single mutant alone (*p* < 0.005 by χ^2^). *n* ≥ 48 for each genotype in all panels.

To determine whether DOS-motif proteins have overlapping functions, we tested whether mutants defective in more than one DOS-motif protein had stronger vulval defects. Loss of either *osm-7* or *dos-1* alone had little or no overt effect on vulval morphology. However, loss of *dos-1* or *osm-7* increased the percentage of *osm-11(lf)* animals with multiple vulval protrusions (Figure 7A). This result is consistent with multiple DOS-motif proteins acting in vulval development.

### OSM-11 Functions with DSL Ligands to Increase Notch Signaling

Classical C. elegans Notch DSL ligands are expressed in VPCs and function redundantly during cell specification [35]. Accordingly, DOS-motif proteins may also function redundantly in VPC specification. One might expect that DSL-domain proteins and DOS-motif proteins would act together to activate Notch signaling. Therefore, loss of a DSL protein should exacerbate *osm-11* developmental defects. *dsl-1* encodes a DSL domain-containing ligand which acts redundantly with two other DSL proteins to activate LIN-12 Notch signaling during vulval development [35]. Because of this redundancy, the *dsl-1(ok810lf)* null allele does not itself cause vulval defects [35]. However, *dsl-1(lf);osm-11(lf)* double mutants had modestly increased phenotypic defects in vulval morphology compared to *osm-11* single mutants. *osm-11(lf)* vulval defects were similarly enhanced by *lag-2(lf)*, which encodes a DSL ligand (Figure 7B). To more precisely assess interactions between *osm-11* and *dsl-1*, the expression of *lip-1p::gfp* in *dsl-1(lf);osm-11(lf)* animals was assessed during VPC fate specification. Eighty-six percent of the double-mutant animals lacked *lip-1p::gfp* up-regulation in either one or both presumptive secondary VPCs, indicating a substantial synergistic loss of secondary fate specification (Figure 7C). This result is consistent with DOS-motif (i.e., OSM-11) and DSL-domain proteins working together to increase LIN-12 Notch signaling.

### OSM-11 Interacts with LIN-12 Extracellular EGF Repeats in a Yeast Two-Hybrid Assay

The cellular nonautonomy of *osm-11*, the similarity of OSM-11 to Notch ligands, the expression pattern of OSM-11, and the genetic implication that *osm-11* functions before or during LIN-12 Notch activation in VPC fate specification collectively suggest that OSM-11 may function as a LIN-12 Notch ligand. We tested the hypothesis that OSM-11 directly interacts with the LIN-12 extracellular domain. Previous studies demonstrated that *Drosophila* and vertebrate DSL ligands bind to the extracellular EGF repeats of Notch receptors. In preliminary studies, we were unable to demonstrate direct binding between OSM-11 and LIN-12 biochemically using a heterologous expression system (unpublished data). Therefore, we turned to the yeast two-hybrid assay to test whether OSM-11 can interact with LIN-12 extracellular EGF repeats. Conventional wisdom suggests that the yeast two-hybrid system is not suitable for testing extracellular protein–protein interactions, especially for domains rich in disulfide bridges (e.g., EGF repeats). However, two-hybrid interactions have been demonstrated between Notch receptors and ligand pairs in other species for which biochemical interactions have been previously validated [38,39,50], as well as for numerous other extracellular proteins [81–86].

**Figure 8:**
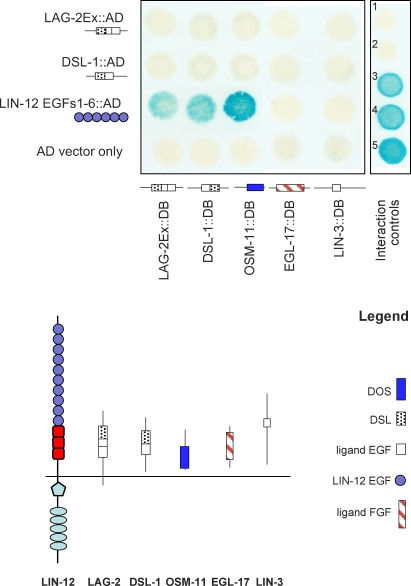
OSM-11 and C. elegans DSL Ligands Interact with LIN-12 Notch Extracellular Domain EGF Repeats in the Two-Hybrid System DSL-1, OSM-11, LAG-2 extracellular domain (LAG-2Ex), EGL-17, or LIN-3 was fused to the GAL4 DNA binding domain (DB); the first six LIN-12 EGF repeats were fused to the GAL4 activation domain (AD). Pairwise interactions were tested with the yeast two-hybrid assay; positive interactions are indicated by blue staining. Both Notch DSL ligands and OSM-11 interacted with LIN-12 EGF repeats, whereas no interaction of LIN-3 EGF or EGL-17 FGF with LIN-12 Notch receptor EGF repeats was detected. LIN-12::DB fusion proteins exhibited strong self-activation (unpublished data); therefore, reciprocal fusions were not tested. Interaction controls are: (1) empty vectors; (2) DB-pRb and AD-E2F; (3) DB-Fos and AD-Jun; (4) Gal4p and pPC86; and (5) DB-DP1 and AD-E2F1.

To validate our yeast two-hybrid approach, we first confirmed that the extracellular domain of LAG-2 [23] and the soluble DSL-domain LIN-12 ligand DSL-1 [35] interact with LIN-12 extracellular EGF repeats 1 through 6 in the two-hybrid assay (Figure 8). To the best of our knowledge, this is the first in vitro evidence that C. elegans DSL ligands may bind directly to LIN-12 Notch. As a negative control and to confirm specificity of the two-hybrid assay, we showed that the unrelated C. elegans ligands LIN-3 (an EGF homolog) and egg laying defective-17 (EGL-17) (an FGF homolog) do not interact with LIN-12 extracellular EGF repeats (Figure 8). The LAG-2 and DSL-1 interactions with LIN-12 in the two-hybrid assay are consistent with previous genetic studies in C. elegans and with biochemical analyses of Notch ligand/receptor interactions in other systems. Ligand-receptor interactions were only assayed using LIN-12 fused to the GAL4 activation domain (AD) as LIN-12 EGF fusion to the DNA-binding domain resulted in strong self-activation in the presence of AD empty vector (unpublished data).

We found that OSM-11 also interacted with LIN-12 extracellular EGF repeats 1 through 6 (Figure 8); OSM-11 did not interact with DSL-1 or LAG-2 ligands. We also confirmed previous studies [50] in which murine DLK1 EGF repeats 1 and 2 containing the DOS motif interacted specifically with murine Notch1 EGF repeats 12 and 13 in the same two-hybrid assay format (unpublished data). The two-hybrid interaction does not necessarily demonstrate that OSM-11 and LIN-12 interact in vivo; however, combined with the genetic interactions, the apical expression pattern of OSM-11 in VPCs, and previous studies of DLK1/Notch interactions, we favor a simple model in which OSM-11 binds directly to LIN-12 Notch EGF repeats. Further biochemical studies will be required to demonstrate DOS-motif protein direct interactions with Notch receptors.

### The Mammalian DOS-Motif Protein DLK1 Can Substitute for OSM-11

Our results suggest that the DOS-motif protein OSM-11 may act as a soluble LIN-12 ligand in C. elegans. This raises the issue of whether other DOS-motif proteins such as DLK1, which has been implicated in Notch signaling in mammalian cells, also acts as soluble Notch ligands. The precise role of DLK1 in mammalian Notch signaling is controversial. To address the function of mammalian DLK1 in Notch signaling, we tested the ability of DLK1 to functionally substitute for OSM-11 in vivo in C. elegans. We found that expression of a soluble, mature DLK1 protein isoform named FA1 [43] in *osm-11(lf)* animals significantly rescued vulval development, consistent with DLK1 protein increasing LIN-12 signaling (Figure 5D). This result suggests that the function of C. elegans DOS-motif proteins is to increase Notch receptor signaling and that the molecular mechanism may be conserved across species.

## Discussion

The data presented herein demonstrate that *osm-11* is required for normal vulval development in C. elegans. *osm-11* encodes a novel cEGF-1 protein that is similar to, but distinct from, previously characterized Notch ligands in vertebrates [53]. OSM-11 contains a previously unidentified protein motif that we have named DOS (Delta and OSM-11) overlapping the EGF motifs. The DOS motif is conserved across species and found in canonical Notch ligands. OSM-11 is a secreted protein that is expressed in VPCs during cell fate specification. Genetic analysis suggests that OSM-11 acts upstream of LIN-12 and that OSM-11 normally increases LIN-12 Notch signaling in vivo. Two-hybrid data and expression on the VPC apical surfaces suggest that OSM-11 may directly bind to the LIN-12 extracellular domain, although additional biochemical studies will be required to further confirm this. Finally, we demonstrated that the mammalian DOS-motif protein DLK1 can partially substitute for OSM-11 in C. elegans vulval development, suggesting that DOS-motif protein function is conserved across species.

**Figure 9:**
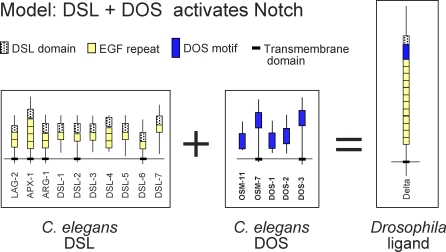
Model: C. elegans DSL and DOS Proteins May Act as Ligands for Notch Receptors Canonical Notch ligands in *Drosophila* contain both DSL domains and DOS motifs as do some vertebrate Notch ligands (e.g., Delta). However, classical Notch ligands from C. elegans and several vertebrate Notch ligands contain a DSL domain, but lack DOS-motif EGF repeats (e.g., LAG-2 or DLL3). The C. elegans proteins characterized in this study (e.g., OSM-11) and the two presumptive vertebrate ligands DLK1 and EGFL9/DLK2 lack DSL domains, but contain DOS motifs. In the simplest model, both a DOS motif and DSL domain are required for coordinated Notch receptor activation. These could act in *cis* in canonical Notch receptors like *Drosophila* Delta or in *trans* in the case of LAG-2 and OSM-11. Overexpression of a “DOS-only” or a “DSL-only” ligand may inhibit Notch receptor activation by competition with canonical ligands containing both a DSL domain and a DOS motif, such as Jagged1 or Delta. This model is consistent with *osm-11(lf)* animals having phenotypic defects usually associated with Notch loss of function. We do not exclude other possible scenarios; see Discussion for details.

Our data suggest a model wherein OSM-11 and C. elegans DSL ligands act together to activate Notch receptors, potentially as a C. elegans bipartite ligand that is functionally equivalent to *Drosophila* Delta or mammalian Jagged1 (Figure 9). Previously described C. elegans DSL ligands such as LAG-2 lack a DOS motif; C. elegans DSL ligands, such as LAG-2, and DOS-motif proteins, such as OSM-11, may both be required to activate LIN-12 Notch receptor signaling in vivo. Classical studies in C. elegans have shown that expression of the APX-1 N-terminus (which contains the DSL domain) is sufficient to activate Notch signaling; however, this is not inconsistent with our model because endogenous DOS-motif proteins were present [23]. Our model is also consistent with previous biochemical and genetic studies that showed the first two EGF repeats of Jagged1 and Delta are critical for high-affinity DSL-domain binding to mammalian Notch receptors and Notch receptor activation [25,54].

Bipartite or heteromeric ligands are relatively rare compared to heteromeric receptors. To our knowledge, bipartite ligands have only been described previously in the immune system. The binding of antigen to complement fragment creates, in effect, a bipartite ligand for antigen receptor as does the binding of an antigenic peptide to a compatible major histocompatibility complex (MHC) subunit. Additionally, and perhaps more pertinently, heterodimeric cytokines have been described in the immune system that bind to cytokine receptors [87]; for example, the interleukin-12 (IL-12) cytokine is composed of p40 and p35, whereas the IL-23 is composed of p40 and p19. Although bipartite ligands are unusual, they are not unprecedented.

Previous studies have shown that the mammalian DOS-motif protein DLK1 acts as a competitive antagonist of ligand Jagged1, a canonical ligand that contains both a DSL domain and DOS motif [50]. Therefore, a plausible alternative model (which takes into account DLK1 antagonism of Jagged1) is that DOS-motif proteins bind to Notch receptors, but function as antagonists of DSL-domain Notch ligands in all species. DOS proteins such as OSM-11 might play a role in maintaining C. elegans Notch receptor levels or localization, although LIN-12 Notch expression is unaltered in animals lacking *osm-11*. Based on our data, we instead favor the simpler model of DOS-motif proteins as activators of Notch receptors acting with DSL proteins. In an independent behavioral analysis (M. Chao, J. Larkins-Ford, T. Tucey, H. Komatsu, and H. Dionne, et al., unpublished data), we also found that OSM-11 activates both LIN-12 and germline proliferation defective-1 (GLP-1) in the adult nervous system to regulate behavior. We speculate that if DLK1 was coexpressed in mammalian systems with a C. elegans DSL-only ligand, then Notch signaling might be increased. Mammalian Delta like 3 (DLL3) and DLL4 ligands contain DSL domains, but not DOS motifs. Biochemical studies have shown that DLL3 inhibits Notch signaling and DLL4 increases Notch signaling in various contexts. It would be useful to examine Notch activation when DLK1 and DLL3 are coexpressed. Clearly, biochemical analyses addressing the role of DOS motifs and DSL domains in Notch receptor activation will be required to discriminate between these two models and to determine the relative contributions of DSL and DOS-motif proteins to Notch signaling.

C. elegans DSL ligands function redundantly, activating LIN-12 Notch during vulval development; loss of any one DSL ligand gene causes mild or no overt defects [35]. Similarly, loss of *osm-11* alone caused only mild defects in vulval morphogenesis, whereas loss of more than one DOS-motif gene resulted in more-severe vulval defects. Like DSL ligands, DOS-motif proteins function semiredundantly to increase Notch signaling in vivo. In addition, genetic analysis suggests that DOS-motif proteins and DSL proteins may act together to regulate Notch receptors. It is possible that Notch receptor activation by ligands during VPC development is robust due to this redundancy. This multifactorial system for regulation of Notch receptors might allow use of individual soluble DOS or DSL proteins in other cell–cell signaling events in other tissues simultaneously.

Defining a role herein for *osm-7* and *osm-11* in Notch signaling suggests that this pathway also plays a previously unsuspected role in osmotic stress response. C. elegans can adapt to increased environmental osmolarity; animals exposed to moderate osmotic stress increase internal osmolyte levels and have altered behavior reminiscent of animals lacking *osm-11* or *osm-7* [51,52]. A role for Notch signaling in osmotic stress has not been reported in any species. The developmental role of Notch signaling in vulval cell fate specification is distinct from the role in osmotic stress response based on data presented here. Further studies will be required to determine whether diffusible DOS proteins act as humoral factors to regulate Notch signaling in multiple tissues to coordinate physiological and behavioral adaptation to osmotic stress.

The diversity of Notch receptors and ligands is remarkable. *C. elegans* has two Notch receptors (*lin-12* and *glp-1*), ten DSL domain proteins that lack DOS motifs [35] and five DOS-motif proteins without DSL domains (this study). Mammals have four Notch receptors, multiple DSL ligands, and two presumptive DOS-motif–only ligands: DLK1 and EGFL9/DLK2. Additional proteins have been suggested to act as Notch ligands in vertebrates [36–40], but invertebrate homologs have not been identified. At least one DSL domain Notch ligand in each vertebrate species we examined (zebrafish, humans, and mice) lacks the conserved DOS motif; these proteins are potentially analogous to C. elegans DSL domain ligands (e.g., LAG-2) that also lack DOS motifs. Soluble Notch ligands are now predicted in all of these species based on this and previous studies. In contrast, *Drosophila* has only one Notch receptor, and the two previously characterized transmembrane *Drosophila* Notch ligands contain both DSL domains and DOS motifs. This heterogeneity of Notch ligands and receptors indicates that the functional relationship between Notch receptors and ligands is highly complex, allowing precise regulation of signaling.

## Materials and Methods

### Characterization of *osm-11.*

The *osm-11(rt68)* mutant allele was identified in a classical genetic screen based on defective chemosensory response and temporarily designated *sel-14* (*suppressor/enhancer of lin-12*-*14*). The *rt68* mutation was mapped to the predicted C. elegans gene F11C7.5 and mutates W177 to a premature stop codon, resulting in premature truncation of translation near the end of the DOS motif. Recent published studies and our analysis herein confirmed that *sel-14(rt68)* is an allele of *osm-11* and has the same amino acid change as the previously identified allele *osm-11(n1604)* [52,88]; therefore, we refer to this gene as *osm-11.* The deletion allele *osm-11(rt142)* was identified by PCR-based screening of a frozen ethylmethane sulfonate (EMS)-mutagenized library of C. elegans strains [89]. The *rt68* and *rt142* alleles had similar phenotypic defects, but the *rt142* deletion allele was more severe. Both *osm-11* alleles are recessive. *osm-11(rt142)* is likely a complete loss-of-function (*lf*) allele and was used exclusively herein. RNAi of *osm-11* was performed by raising N2 animals on a lawn of bacteria expressing *osm-11* double-stranded (dsRNA). Other than morphological defects, vulva perturbations, and consequent egg-laying defects, *osm-11(rt142lf)* animals are overtly normal in locomotion, male mating, and reproduction, although their growth rate is slightly slower and they are slightly smaller than wild-type animals. *osm-11* animals frequently had ventral protrusions posterior to the anus. Postanal swelling is frequently associated with bacterial infections, but swelling occurs in uncontaminated *osm-11* animals raised on standard OP50 bacteria. Gonad morphology was subtly altered in *osm-11* animals but was not further characterized here. *osm-11* loss of function alters *glp-1* germline proliferation defects (M. Chao, J. Larkins-Ford, T. Tucey, H. Komatsu, and H. Dionne, et al, unpublished data). *osm-11(rt142)* animals are osmotic stress resistant and are motile on 500 mM NaCl NGM plates, consistent with previously published phenotypes of *osm-11(n1604)* [52].

### Strains and genetics.

Gain-of-function *lin-12* alleles used herein included the constitutive dominant allele *lin-12(n137gf)* and the cold-sensitive recessive gain-of-function allele *lin-12(n137n460gfcs)*. Results from homozygous *lin-12(n137)* and heterozygote *lin-12(n137)/+* animals were pooled in Figure 6. The *lin-12(n941)* null allele was maintained by balancing over either *qC1* containing *qIs26* [*rol-6(d), lag-2::gfp*] or over *unc-32(e189)*. Genetic epistasis of *osm-11* with *lin-12(n941)* was assessed using homozygous *lin-12(lf)* progeny of *lin-12(lf)/unc-32(e189)* animals. A fraction of animals were singled as larvae and subsequently scored for vulval morphology and genotype. *lin-12(n941)* animals were always sterile regardless of *osm-11* status; *lin-12(n941)/unc-32* animals lacked protruding vulva and yielded *unc-32* progeny regardless of *osm-11* status.

The deletion alleles *osm-7(tm2256)* and *dos-1(ok2398)* were generated by the C. elegans gene knockout consortia. The *osm-7(tm2256)* deletion removes the first part of the DOS motif and eliminates an exon splice site, resulting in a predicted frame shift after amino acid 200 with premature truncation after translation of 21 amino acids. The *dos-1(ok2398)* allele is a 1.7-kb deletion that removes the initiator methionine and the first five exons, including the DOS motif. All deletion alleles were backcrossed at least four times prior to analysis. Double-mutant analysis in Figure 7 was performed in an *ayIs4* genetic background. Other alleles used in this study include *lag-2(sa37)* and *dsl-1(ok810)*.

### Analysis of VPC fate specification.

Pn.p cells and descendents were identified by differential interference contrast (DIC) imaging on a Zeiss Axioskop2. The transgenic arrays used for VPC fate analysis were: *ayIs4 [egl-17p::gfp]*, *syIs107 [lin-3p::gfp]*, *oyIs31 [lin-11p::gfp]*, and *zhIs4 [lip-1p::gfp]* [58,60,63,90]. Animals were scored at the Pn.p and Pn.px stages for *egl-17p::gfp* and *lip-1p::gfp*, but only at the Pn.pxx stage for *lin-11p::gfp*. Rearing on 400 mM NaCl NGM plates dramatically slows growth and results in partially penetrant embryonic and larval lethality. In less than 10% of all animals raised under these conditions, Pn.p cells/descendents could not be identified by DIC; these animals were excluded from the analysis. *oyIs31* animals were nonviable on 400 mM NaCl NGM plates

### Immunohistochemistry.

Polyclonal antisera specific to OSM-11 were raised in rabbits using the C-terminal peptide YSKCTMFTPVQY (Sigma-Genosys) and was used as a 1:200 dilution of unpurified sera. OSM-11 immunoreactivity was detected in larval and adult animals in paraformaldehyde-fixed wild-type animals, but not in *osm-11(rt142lf)* animals (unpublished data). Eggs were not examined, and no immunoreactivity in germ cells was observed. OSM-11 was detected at the junction of the presumptive vulva and uterus of L4 larvae and in the spermatheca of late-larval and adult animals. OSM-11 mRNA localization by in situ hybridization is consistent with expression in VPCs and hypoderm in young larvae and in seam cells in adult animals (see NEXTDB, http://nematode.lab.nig.ac.jp/db2/ShowCloneInfo.php?clone=59g10; Y. Kohara, personal correspondence).

### Molecular biology.

Plasmids and cloning details are available upon request. Transgenic strains were generated by microinjection with plasmids of interest at 20 to 50 ng/μl. Transgenesis coinjection markers were pJM#67 *elt-2::gfp* [91], pPD48.33 *myo-2::gfp* [92], or phenotypic rescue of *pha-1(e2123)* using pBX#1 [93]. The *osm-11* cDNA clone was obtained by PCR from the Vidal laboratory ORFeome cDNA library [94] and agrees exactly with the predicted sequence in WormBase and at NCBI. *osm-11* cDNA constructs used herein for rescue contained the *unc-54* 3′ UTR, whereas genomic rescue clones contained the *osm-11* 3′ UTR. Although *osm-11* vulval defects are substantially rescued by both types of constructs, we cannot rule out transcriptional regulation by the *osm-11* 3′ UTR. Multiple transgenic lines were scored for each transgenic experiment; results were substantially equal for each transgenic line and were pooled by construct. The soluble *lag-2* construct was previously described and fully rescues a *lag-2* mutant [23]. Mammalian DLK1 has multiple splice forms yielding soluble and membrane-bound isoforms. Proteolysis of membrane-bound DLK1 yields the soluble protein originally known as fetal antigen 1 (FA1). A murine DLK1 cDNA fragment that encodes the DLK1 FA1 protein isoform was used in C. elegans rescue experiments and was expressed ubiquitously using the *hsp-16* promoter.

### Bioinformatics.

C. elegans and C. briggsae homologs of OSM-11 were identified by BLAST analysis against genomic sequences and predicted genes at NCBI and WormBase. A short, common motif was identified manually and used to search for similar proteins using Pattern Search at the Swiss Institute for Experimental Cancer Research (ISREC) (http://myhits.isb-sib.ch/cgi-bin/pattern_search). A subset of Notch ligands was identified. DLK1 and *Drosophila* Delta proteins were manually compared to C. elegans and C. briggsae homologs of OSM-11 and used to generate the final DOS-motif consensus (Figure 2). Proteins were aligned using ClustalW at ISREC (http://myhits.isb-sib.ch/cgi-bin/clustalw). The proteins identified are known Notch ligands except for mouse DLK1, *Drosophila* C901, and human EGFL9. *Drosophila* C901 contains a signal peptide, a DSL domain, and EGF repeats, but has not been well characterized [56]. DLK1 and EGFL9 do not contain DSL domains, but do contain signal peptides and EGF repeats. Given that all previously identified DSL domains are located between the signal peptide sequence and the EGF repeats, we conclude that DLK1 and EGFL9 do not contain DSL domains. It is interesting to note that many classical Notch ligand genes contain an intron immediately after the DSL domain.

T05D4.4 and ZK507.4 (OSM-7 and DOS-1, respectively) predicted C. elegans proteins are partially confirmed by existing cDNAs and are conserved in C. briggsae. A cDNA fragment containing predicted C. elegans K10G6.2 (*dos-2*) exons was successfully amplified from a cDNA library by the Vidal ORFeome project; the K10G6.2 predicted protein is also conserved in C. briggsae. The C. briggsae homologs of C. elegans proteins are CBG18238 for T05D4.4, CBG18440 for K10G6.2, CBG06935 for ZK507.4, and CBG15929 for F11C7.5. The new prediction for K02F3.7/DOS-3 has been submitted to WormBase; the C. briggsae homolog is CBP19746. All of these C. briggsae proteins are predicted to have signal peptide sequences. Proteins in D. melanogaster, C. elegans, C. briggsae, *Homo sapiens*, *Danio rerio*, and *Mus musculus* that contain the DOS motif are (amino acids): tr:A1L1P2_DANRE/224–274, tr:A1C3M9_DANRE/228–278, sw:DLL1_HUMAN/226–276, sw:DLL1_MOUSE/225–275, tr:A4V346_DROME/231–279, sw:DLLB_DANRE/208–258, tr:Q9VZ44_DROME/212–262, tr:Q925U3_MOUSE/26–76, NP_003827/26–76, sw:EGFL9_HUMAN/29–79, sw:Q8K1E3 EGFL9/29–79, tr:A1A3Y8_DANRE/235–285, tr:A1A3Y7_DANRE/231–281, sw:JAG1_HUMAN/234–284, sw:JAG1_MOUSE/234–284, sw:JAG2_MOUSE/245–295, sw:JAG2_HUMAN/245–295, tr:Q90Y55_DANRE/237–287, sw:SERR_DROME/284–335, tr:O45750_CAEEL/205–253, tr:Q60YH7_CAEBR/205–253, tr:Q21149_CAEEL/127–175, tr:Q60JE9_CAEBR/385–433, sw:YOO4_CAEEL/130–179, tr:Q614N0_CAEBR/135–180, tr:O45346_CAEEL/136–181, and tr:Q60Y06_CAEBR/130–177.

DOS-motif proteins were clustered using CLUSTALW in the MegAlign package (Lasergene) with an identity matrix and the following default parameters: gap penalty 20.0, gap length penalty 0.2, delay divergent sequences 30%, and DNA transition weight 0.5. The N- and C-terminal boundaries of the amino acid sequences used for the alignment began at the first cysteine residue of the first EGF repeat, and ended at the cysteine residue immediately preceding the conserved CXC motif of the second EGF repeat. The only exceptions to this were the sequences used for *Mm*Jagged1 and *Hs*Jagged1; in these proteins, a gap between EGF repeats 1 and 2 contained cysteine and tryptophan residues that followed the spacing of the DOS motif consensus sequence but were clearly not part of EGF repeat 2. Amino acid sequence from the gap instead of from EGF repeat 2 was used for these two proteins. Two outgroups were used in the alignment: EGF repeats 1 and 2 from *Ce*APX-1 and *Ce*LAG-2, which lack the SELCT motif and have been previously shown to be phylogenetically distinct from EGF repeats 1 and 2 of other DSL ligands [27]; and EGF repeats 3 and 4 (EGF3–4) of selected DOS motif–containing proteins (using the same N- and C-terminal boundaries as above), as examples of canonical EGF repeats. *Dm*Serrate EGF repeat 4 contains a phylogenetically unique insertion; for the purposes of sequence alignment, amino acids 407–470 were deleted [27]. Accession numbers used are: *Ce*T05D4.4, O45750; *Ce*zk507.4, P34636; *Ce*SEL-14, O45346; *Ce*K10G6.2, O16627; *Ce*APX-1, P41990; *Ce*LAG-2, P45442; *Dr*DeltaA, AAC41249; *Dr*DeltaB, AAH76414; *Dr*DeltaD, Q8UWJ4; *Dr*Jagged1, Q90Y57; *Dr*Jagged2, CAH69088; *Dr*SerrateB, AAC98354; *Dm*Delta, P10041; *Dm*Serrate, P18168; *Dm*C901, CAA72010; *Hs*Dll1, O00548; *Hs*Egfl9, Q6UY11; *Hs*Jagged1, P78504; *Hs*Jagged2, Q9Y219; *Mm*Dlk1, NP_034182; *Mm*Dll1, Q61483; *Mm*Jagged1, Q9QXX0; and *Mm*Jagged2, Q9QYE5. Species designations are: *Ce*, Caenorhabditis elegans; *Dr*, Danio rerio; *Dm*, Drosophila melanogaster; *Hs*, Homo sapiens; *Mm*, Mus musculus.

## Abbreviations

AC: anchor cell
AD: activation domain
APX: anterior pharynx defective
DLK: Deltalike
DLL: Delta like
DOS: Delta and OSM-11
DSL: Delta, Serrate, and LAG-2
EGF: epidermal growth factor
EGFL: EGFlike
FA1: fetal antigen 1
GFP: green fluorescent protein
LAG: Lin and Glp
LIN: lineage defective
Muv: multivulva
RNAi: RNA interference
VPC: vulval precursor cell
VU: vulval uterine
